# Evidence That Humans Metabolize Benzene via Two Pathways

**DOI:** 10.1289/ehp.0800510

**Published:** 2009-02-19

**Authors:** Stephen M. Rappaport, Sungkyoon Kim, Qing Lan, Roel Vermeulen, Suramya Waidyanatha, Luoping Zhang, Guilan Li, Songnian Yin, Richard B. Hayes, Nathaniel Rothman, Martyn T. Smith

**Affiliations:** 1 School of Public Health, University of California at Berkeley, Berkeley, California, USA; 2 School of Public Health, University of North Carolina at Chapel Hill, Chapel Hill, North Carolina, USA; 3 National Cancer Institute, National Institutes of Health, U.S. Department of Health and Human Services, Bethesda, Maryland, USA; 4 Institute for Risk Assessment Sciences, Utrecht University, Utrecht, The Netherlands; 5 Institute of Occupational Health and Poison Control, Chinese Center for Disease Control and Prevention, Beijing, China

**Keywords:** benzene, biomonitoring, cancer risk, cytochrome P450, metabolism

## Abstract

**Background:**

Recent evidence has shown that humans metabolize benzene more efficiently at environmental air concentrations than at concentrations > 1 ppm. This led us to speculate that an unidentified metabolic pathway was mainly responsible for benzene metabolism at ambient levels.

**Objective:**

We statistically tested whether human metabolism of benzene is better fitted by a kinetic model having two pathways rather than one.

**Methods:**

We fit Michaelis-Menten-like models to levels of urinary benzene metabolites and the corresponding air concentrations for 263 nonsmoking Chinese females. Estimated benzene concentrations ranged from less than 0.001 ppm to 299 ppm, with 10th and 90th percentile values of 0.002 ppm and 8.97 ppm, respectively.

**Results:**

Using values of Akaike’s information criterion obtained under the two models, we found strong statistical evidence favoring two metabolic pathways, with respective affinities (benzene air concentrations analogous to *K_m_* values) of 301 ppm for the low-affinity pathway (probably dominated by cytochrome P450 enzyme 2E1) and 0.594 ppm for the high-affinity pathway (unknown). The exposure-specific metabolite level predicted by our two-pathway model at nonsaturating concentrations was 184 μM/ppm of benzene, a value close to an independent estimate of 194 μM/ppm for a typical nonsmoking Chinese female. Our results indicate that a nonsmoking woman would metabolize about three times more benzene from the ambient environment under the two-pathway model (184 μM/ppm) than under the one-pathway model (68.6 μM/ppm). In fact, 73% of the ambient benzene dose would be metabolized via the unidentified high-affinity pathway.

**Conclusion:**

Because regulatory risk assessments have assumed nonsaturating metabolism of benzene in persons exposed to air concentrations well above 10 ppm, our findings suggest that the true leukemia risks could be substantially greater than currently thought at ambient levels of exposure—about 3-fold higher among nonsmoking females in the general population.

Benzene is an important industrial chemical that is also present in gasoline, engine exhausts, wood smoke, and tobacco smoke [International Agency for Research on Cancer (IARC) 1989]. In fact, benzene is truly ubiquitous in the environment, with air concentrations ranging from parts per billion in rural and urban settings to parts per million in some workplaces (IARC 1989; Wallace 1996). This is worrisome because benzene causes leukemia and probably other lympho-hematopoietic cancers in humans (Hayes et al. 1997; Infante et al. 1977; Rinsky et al. 1987), and there is evidence that benzene is hematotoxic at levels < 1 ppm (3.2 mg/m^3^) (Lan et al. 2004), the current permissible exposure limit in the United States (Occupational Safety and Health Administration 1987). Benzene also produces malignant tumors at multiple sites in rodents (Huff et al. 1989; Maltoni et al. 1989).

The toxicology of few chemicals has been pursued as vigorously as that of benzene. A PubMed search of the keywords “benzene” and “toxicity” returned more than 2,700 publications. Reports of benzene’s propensity to damage human blood-forming tissues emerged as early as 1897 (Santesson 1897), and the first evidence of its leukemogenicity was published in 1928 (Delore and Borgomano 1928).

Although benzene must be metabolized to exert toxicity, the metabolism is complex, and particular roles played by the various metabolites have not been fully elucidated (Ross 1996; Smith 1996; Snyder 2002). As shown in Figure 1, benzene is oxidized by cytochrome P450 (CYP) enzymes to benzene oxide, which exists in equilibrium with its tautomer oxepin. Spontaneous rearrangement of benzene oxide produces phenol that is either excreted or oxidized by CYP enzymes to hydroquinone, which is excreted or oxidized to 1,4-benzoquinone. Other major metabolites include catechol (which can be oxidized to 1,2-benzoquinone), after hydrolysis of benzene oxide and aromatization of benzene dihydrodiol, and *E*,*E*-muconic acid (hereafter “muconic acid”), after oxidation of oxepin and ring opening. Reaction between benzene oxide and glutathione, possibly mediated by glutathione-*S*-transferases, can produce the minor metabolite *S*-phenylmercapturic acid (SPMA). For humans exposed to benzene at air concentrations between 0.1 and 10 ppm, phenol represents 70–85% of urinary benzene metabolites, whereas hydroquinone, muconic acid, and catechol each represent 5–10%, and SPMA represents less than 1% (Kim et al. 2006b).

Benzene oxide, the benzoquinones, muconaldehydes, and benzene diol epoxides (formed from CYP oxidation of benzene dihydrodiol) are electrophiles that readily react with peptides and proteins (Bechtold et al. 1992; Henderson et al. 2005; McDonald et al. 1993; Waidyanatha et al. 2005) and can thereby interfere with cellular function (Smith 1996). Reactions between 1,4-benzoquinone and critical nucleophilic loci in topoisomerase II, an important enzyme involved in DNA replication and maintenance, are mentioned as likely key events in the cascade of effects related to benzene’s ability to damage chromosomes (Chen and Eastmond 1995; Lindsey et al. 2004).

Although there is undoubtedly a causal link between benzene exposure and leukemia, the shape of the exposure–response relationship is controversial, particularly at low doses. Indeed, when considering regulatory actions, litigation, and potential cleanup costs in the billions of dollars, this uncertainty represents a major challenge for environmental toxicology and epidemiology. Recent action by the U.S. Environmental Protection Agency (EPA) to reduce cancer risks from mobile sources underscores this point (U.S. EPA 2007). In justifying its decision to lower the benzene content of gasoline, the U.S. EPA cited studies pointing to supralinear (greater-than-proportional) production of benzene-related protein adducts at air concentrations < 1 ppm (Rappaport et al. 2002, 2005). Such behavior would likely result from saturation of the metabolism of benzene to benzene oxide/oxepin. Because the U.S. EPA had previously assumed that human benzene metabolism proceeded according to nonsaturating (first-order) kinetics at exposure concentrations well above 10 ppm, saturation of metabolism below 1 ppm “could lead to substantial underestimation of leukemia risks” in the general population (U.S. EPA 2007).

Because the dose-related metabolism of benzene had been poorly characterized in humans, we conducted a detailed investigation of urinary metabolite levels among 386 workers in Tianjin, China, for whom individual levels of benzene in air had been documented on the days of urine collection (Kim et al. 2006a). We observed that metabolite levels were significantly affected by benzene exposure as well as sex, age, smoking, single nucleotide polymorphisms of prominent metabolizing genes, and gene–environment and gene–smoking interactions (Kim et al. 2006b, 2007). Intriguingly, the exposure-specific production of major metabolites (phenol, muconic acid, hydroquinone, and catechol, in micromolar per parts per million benzene) decreased continuously with estimated exposure levels over the range of 0.03–88.9 ppm, with the most pronounced decreases occurring at benzene concentrations < 1 ppm (Kim et al. 2006a).

Given the unanticipated decline in exposure-specific metabolism at air concentrations < 1 ppm, we speculated that benzene metabolism might be governed by two saturable pathways, one operating primarily > 1 ppm, and the other primarily at lower concentrations. To test this conjecture, we combined exposure and urinary metabolite data from two studies of Chinese workers, representing 428 subjects exposed to benzene at estimated levels ranging from < 0.001 ppm to > 300 ppm (Kim et al. 2006a; Waidyanatha et al. 2004). Because sex and smoking had previously been shown to affect benzene metabolite levels at a given exposure concentration in this population, we restricted our analyses to 263 nonsmoking females with valid data (estimated benzene exposure range, < 0.001–299 ppm; we removed one previously identified outlier). Because metabolic processes follow saturable (Michaelis-Menten) kinetics, we fit Michaelis-Menten-like models to the molar levels of urinary benzene metabolites (the sum of phenol, muconic acid, hydroquinone, catechol, and SPMA) in these subjects and used statistical fit criteria derived from information theory to weigh the evidence favoring the two models. Our results provide strong statistical evidence favoring two saturable pathways, one operating primarily at concentrations < 1 ppm and the other primarily at concentrations > 1 ppm.

## Materials and Methods

### Study population and air and biological monitoring

Study subjects derive from two cross-sectional molecular epidemiology studies of Chinese benzene-exposed and control workers carried out in Shanghai in 1992 (Rothman et al. 1996, 1998; Waidyanatha et al. 2004) and in Tianjin in 2000–2001 (Kim et al. 2006a; Lan et al. 2004; Vermeulen et al. 2004). Subject enrollment and interview procedures, exposure assessment methods, and urinary metabolite measurements in these two studies were carried out by the same group of investigators using the same procedures. All subjects gave informed written consent to act as human subjects, and all applicable approvals were obtained. Workers with occupational exposure to benzene were employed in factories where benzene was present, and control workers were exposed to airborne benzene in the general environment, as determined by measurement of urinary benzene (Tianjin controls only) (Kim et al. 2006a). Among non-smoking females, 159 benzene-exposed and 84 control subjects had complete data. Table 1 shows summary statistics regarding benzene exposure, age, body mass index, and weight.

The methods of sampling air and urine have been reported previously (Kim et al. 2006a; Lan et al. 2004; Vermeulen et al. 2004; Waidyanatha et al. 2001). Briefly, we matched personal full-shift air measurements with postshift urine samples from exposed and control workers. Statistical analyses used the sum of the molar concentrations of phenol, hydroquinone, catechol, muconic acid, and SPMA (hereafter “total metabolites”). Three subjects were missing measurements of SPMA. Because SPMA rarely exceeds 1% of total metabolites, we estimated levels of total metabolites for these three subjects as the sum of phenol, hydroquinone, catechol, and muconic acid. Of the 263 subjects in this analysis, 243 had repeated measurements of air and urine, making a total of 391 matched air/urine samples. Subjects with repeated measurements had a median of three paired air and urine samples (range, 2–4).

Benzene was measured in air using passive personal monitors (Organic Vapor Monitors, model 3500; 3M, St. Paul, MN, USA) followed by solvent desorption and gas chromatography (GC) with flame ionization detection (Vermeulen et al. 2004). A total of 161 air measurements were either missing or below the limit of detection (normally 0.2 ppm). We predicted air concentrations for these censored and missing air samples from the corresponding levels of urinary benzene, as described previously (Kim et al. 2006a). The estimated median exposure to benzene among the non-smoking female subjects was 0.644 ppm, with 10th and 90th percentile values of 0.002 ppm and 8.97 ppm, respectively (Table 1).

We determined urinary benzene by GC-mass spectrometry (MS) using head-space solid-phase microextraction according to the method of Waidyanatha et al. (2001). Urinary phenol, hydroquinone, catechol, muconic acid, and SPMA were measured as trimethylsilyl derivatives by GC-MS, after acid hydrolysis of urine to release conjugates, according to Waidyanatha et al. (2004). Quantification of all urinary analytes was based on peak areas relative to the corresponding isotopically labeled internal standards.

The Shanghai workers were exposed to essentially pure benzene, whereas all Tianjin workers were coexposed to toluene, and some were exposed to low levels (< 5 ppm) of other volatile organic compounds (Vermeulen et al. 2004). We previously found no significant effects of toluene coexposure on levels of benzene metabolites in the Tianjin workers (Kim et al. 2006b).

### Michaelis-Menten-like models

We examined relationships between levels of total metabolites and the corresponding air concentrations of benzene using nonlinear regression models (implemented with the SAS procedure NLIN; SAS Institute Inc., Cary, NC, USA). To consider saturable metabolism, we invoked Michaelis-Menten-like models, where the enzymatic velocity (*v*) was replaced with the level of total metabolites (*Y*) (micromolar), and the substrate concentration with the air concentration of benzene (*X*) (parts per million). The asymptotically maximum level of *Y* (designated *Y*_max_) is analogous to *v*_max_, and the benzene concentration *X* at which *Y* = *Y*_max_/2 (designated *X*_50_, in parts per million) is analogous to *K_m_*. We also assumed a background level of total metabolites *Y*_0_ (micromolar) due to endogenous and dietary sources of the individual compounds, particularly phenol, hydroquinone, and catechol (Kim et al. 2006a; McDonald et al. 2001). Given our hypothesis that two saturable pathways governed benzene metabolism rather than one, we fit two Michaelis-Menten-like models to the data, one having a single metabolic pathway and the other having two pathways that competed for access to benzene (*X*). The following expressions define the two models:


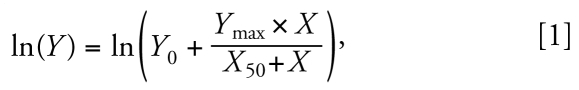



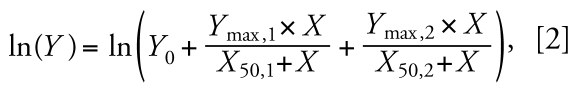


where the subscripts 1 and 2 in Equation 2 refer to the first and second metabolic pathways, respectively. We will refer to Equation 1 as the “one-pathway model” and to Equation 2 as the “two-pathway model.” Also, because the affinity of a given enzyme pathway is indicated by *X*_50_ (the benzene air concentration at which *Y* is half-maximal, analogous to *K_m_*), we define pathway 1 as the low-affinity pathway and pathway 2 as the high-affinity pathway, which is to say that *X*_50,2_< *X*_50,1_.

Each data pair (*Y*, *X*) represents the post-shift total metabolite concentration and the corresponding full-shift benzene air concentration for a given subject. For subjects with repeated measurements (*n* = 243), we used estimated geometric means of air and total metabolite levels. Given the highly skewed and heteroskedastic levels of benzene in air and total metabolites, natural log transforms of *Y* and the independent variables were used for regression analyses. Initial values for *Y*_0_, *Y*_max_, *X*_50_, *Y*_max,1_, and *X*_50,1_ were estimated from scatter plots of benzene metabolite levels versus benzene exposure. Because we defined metabolic pathway 2 as having higher affinity and lower capacity than metabolic pathway 1, we assigned initial values to *Y*_max,2_ ≪ *Y*_max,1_ and to *X*_50,2_ ≪ *X*_50,1_. However, the final model was not sensitive to initial values of these parameters. The estimated kinetic parameters were used to construct overall profiles and partial profiles for the one-pathway and two-pathway models. Uncertainties in the parameters estimated under Equation 1 and Equation 2 were evaluated via bootstrap resampling with replacement (*n* = 1,000, implemented with the SAS macro %boot). We used bootstrap results to estimate median, 2.5th, and 97.5th percentile values for each kinetic parameter in Equations 1 and 2 as well as the ratios *Y*_max,1_:*X*_50,1_ and *Y*_max,2_:*X*_50,2_, which represent maximum rates of metabolism (micromole per parts per million) for the two pathways (analogous to *v*_max_/*K_m_*), respectively.

### Weights of evidence for models

In judging the weight of evidence favoring either the one-pathway model or two-pathway model as a depiction of the true metabolism for benzene (i.e., “truth”), we employed criteria derived from information theory that offer advantages over hypothesis testing in selecting among candidate models derived from scientific considerations (Burnham and Anderson 2002). Because the models represented by Equations 1 and 2 are nested, and the number of observations (*n* = 263) exceeds 40 times the number of model parameters (three or five), Akaike’s information criterion (AIC) provides an appropriate means for judging the better fit; that is, the model with the smaller AIC is better. The difference in AIC values (Δ*_i_*) between the *i*th model and the best model and the associated Akaike weights,


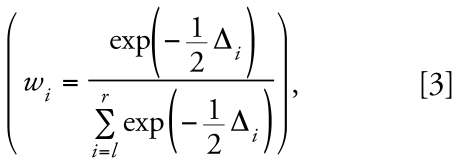


(where *r* is the number of models for comparison; *r* = 2 here) provide information about the weights of evidence supporting the competing models. By definition, the best model (i.e., the one with the smallest AIC) has a value of Δ_min_ = 0. Therefore, *w_i_* varies between exp(−Δ*_i_*/2) and 1 and represents the odds that the *i*th model is, in fact, the best depiction of truth, given the available data. Thus, a model having *w_i_* > 0.9 offers a compelling depiction of truth with the data at hand, and a model with *w_i_* < 0.1 offers a poor depiction of truth (Burnham and Anderson 2002).

All statistical analyses were performed using SAS software for Windows version 9.13 (SAS).

## Results

Figure 2 shows a scatterplot of total metabolite levels for the 263 subjects versus the shift-long air concentration of benzene on the day of urine collection. At benzene concentrations < 0.1 ppm, the data primarily reflect background sources of the metabolites. The effect of benzene exposure on metabolite production becomes increasingly apparent at air concentrations > 1 ppm, and at levels > 100 ppm there is evidence that metabolite levels are approaching a plateau. There is a roughly 5-fold range of metabolite levels among nonsmoking females at a given air concentration of benzene.

### Estimated parameters for the Michaelis-Menten-like models

Also shown in Figure 2 are the mean trends for metabolite levels predicted from the parameters of the one-pathway model (dashed curve) and the two-pathway model (solid curve). Although both models offer qualitatively similar fits to the data in log scale, there are important quantitative differences, as shown by the estimated parameters in Table 2 and the ratios *Y*_max,1_:*X*_50,1_ and *Y*_max,2_:*X*_50,2_. Thus, the question of which model provides a better fit to the data is extremely important. Because the AIC values were –313.755 for the one-pathway model (Equation 1) and −319.320 for the two-pathway model (Equation 2), resulting in a difference of −313.755 − (−319.320) = 5.473, we conclude that the two-pathway model provides a much better fit to the data. (Note that the weight of evidence supporting the two-pathway model depends strictly upon the difference in AIC values between the two models rather than the relative magnitudes of the AIC values under the two models.) this conclusion is supported by the Akaike weights of *w*_1_ = 0.061 and *w*_2_ = 0.939 for the one-pathway and two-pathway models, respectively, which indicate that, given the available data, Equation 2 offers a statistically robust depiction of the true benzene metabolism.

Table 2 also summarizes results from bootstrap resampling of the data under the one-pathway and two-pathway models. In both cases, the medians of the bootstrap realizations are very similar to the parameters estimated by the original models. The uncertainties in parameter estimates are indicated by the range between the 2.5th and 97.5th percentile values from the 1,000 bootstrap samples in each case.

To visualize predicted metabolite levels free of background effects, the curves in Figure 3 represent the background-adjusted benzene metabolite levels, given by (Ŷ_x_−Ŷ_0_) where Ŷ_x_ and Ŷ_0_ represent the predicted levels of benzene metabolites at benzene concentrations *X* and 0 ppm, respectively, using the estimated parameters from Equation 1 (blue curve) and Equation 2 (black curve) from the original regressions (Table 2). Metabolite levels predicted from the two models are similar for benzene exposures in the range of 3–100 ppm but differ substantially for lower exposure concentrations.

### Exposure-specific metabolism of benzene at parts per billion exposure levels

The surrogate for the maximum metabolic rate of benzene (*v*_max_/*K_m_*) in our analyses is the exposure-specific level of benzene metabolites (micromolar per parts per million) produced at nonsaturating (parts per billion) air concentrations. Under the one-pathway model (Equation 1), this exposure-specific metabolite level would be *Y*_max_/*X*_50_ = 68.6 μM/ppm, whereas under the two-pathway model (Equation 2) it would be the sum *Y*_max,1_/*X*_50,1_ = 48.7 μM/ppm plus *Y*_max,2_/*X*_50,2_ = 135 μM/ppm, or 184 μM/ppm (Table 2).

Based on AIC values given above, the fit of the two-pathway model was superior to that of the one-pathway model. Thus, we sought independent confirmation that the exposure-specific metabolite level predicted under Equation 2 (184 μM/ppm) was, indeed, more reasonable than that from Equation 1 (68.6 μM/ppm) at parts per billion levels of exposure. In order to estimate metabolite levels for a typical non-smoking Chinese female exposed to benzene at parts per billion levels, information is required regarding the rates of inhalation and urine production and the fractions of the inhaled dose retained in the lung and excreted as urinary metabolites. The first two pieces of information are relatively straightforward because a typical Chinese female working at light exercise should have a breathing rate of 0.863 m^3^/hr (Yu et al. 2001) and should produce 63.8 mL of urine per hour (Fitzgerald and Brubaker 2003). However, lung retention of benzene at parts per billion exposure levels is less obvious because relatively few studies have reported both inhaled and exhaled breath concentrations of benzene among persons exposed at parts per billion levels. Based on the original data from another study conducted in our laboratory among 19 nonsmoking garage mechanics (Egeghy et al. 2002), we estimated a median value of 73% lung retention of benzene (range, 45–86%) based on geometric mean inhaled air (range, 7–204 ppb) and breath concentrations (range, 1.9–54.6 ppb) for three independent measurements per subject (breath levels were measured immediately after 4-hr personal measurements of inhaled air). Regarding the fractional excretion of benzene metabolites at parts per billion levels (free of background effects), we relied upon the estimated median excretion value of 48% among four subjects experimentally exposed to 40 ppb ^13^C-benzene for 2 hr (Weisel et al. 2003). Using these values, the exposure- specific metabolite level for a typical nonsmoking female exposed to benzene for several hours at parts per billion levels should be


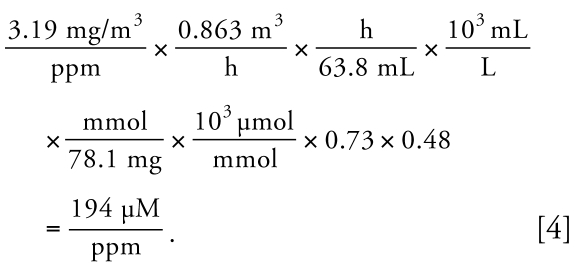


Clearly, this value of 194 μM/ppm (Equation 4) is similar to that of 184 μM/ppm predicted under our two-pathway model and is substantially larger than that of 68.6 μM/ppm predicted under our one-pathway model.

### Percentages of benzene metabolized by the two pathways

The two solid curves in Figure 3A represent the background-adjusted partial metabolite trends corresponding to the two metabolic pathways, again based on the original regression. These partial trends indicate that pathway 1 began to saturate at a benzene concentration of about 200 ppm, and that pathway 2 began to saturate at concentrations < 0.1 ppm and was near full saturation at 1 ppm. Figure 3B shows relative percentages of benzene metabolites attributable to the two metabolic pathways at increasing levels of benzene exposure. At air concentrations < 0.04 ppm, about 73% of benzene metabolites derived from pathway 2. As the benzene concentration increased, the proportion of benzene metabolites derived from pathway 2 dropped to 50% at about 1 ppm and to 10% at about 12 ppm. Above 12 ppm, pathway 1 accounted for virtually all metabolism of benzene.

## Discussion

We previously used regression splines to characterize nonlinear relationships between levels of individual benzene metabolites and air exposures in 386 Chinese workers (Kim et al. 2006b, 2007). Although spline models offered useful basis functions for evaluating the effects of physiologic, lifestyle, and genetic factors upon benzene metabolism, they shed little light on the underlying toxicokinetics, other than to suggest some inconsistency with the notion that benzene was metabolized by a single saturable pathway. This led us to question whether an unrecognized high-affinity/low-capacity pathway might contribute substantially to human benzene metabolism at air concentrations < 1 ppm. Previous investigators noted dose-related differences in excretion patterns of muconic acid and/or hydroquinone between rats and mice (Medinsky et al. 1989, 1996; Sabourin et al. 1989) and between strains of mice (Witz et al. 1990), which they attributed variously to competition across benzene and its metabolites for the same enzymes, competition across phase I and phase II pathways for elimination of individual metabolites, and different activities of benzene-metabolizing enzymes across species or strains. Also, Yu and Weisel (1996a, 1996b) noted that humans exposed to subparts per million levels of benzene absorbed significantly larger doses and excreted larger proportions of the absorbed dose as muconic acid than did those exposed at parts per million levels. However, to our knowledge no one has previously suggested that dose-related behavior might arise, at least in part, from the presence of two benzene-metabolizing pathways having vastly different affinities.

**Figure 1:**
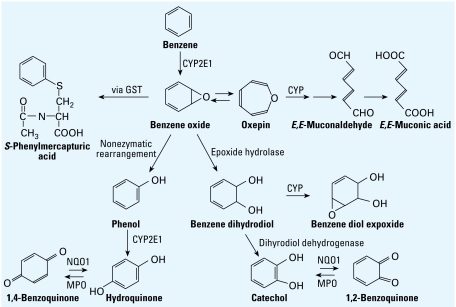
Simplified metabolic scheme for benzene showing major pathways and metabolizing genes. GST, glutathione-*S*-transferase; NQO1, NAD(P)H:quinone oxidoreductase 1; MPO, myeloperoxidase; CYP2E1, cytochrome P450 2E1.

To determine whether human benzene metabolism involves two saturable pathways, we examined the fits of two Michaelis-Menten-like models (Equations 1 and 2) with air and total metabolite data from 263 nonsmoking female subjects, exposed to estimated benzene air concentrations ranging from < 1 ppb to 299 ppm. Because we wished to model the quantitative metabolism of benzene per se, we focused on total metabolites rather than individual benzene metabolites, which reflect additional saturable processes (see Figure 1). In doing so, we modified the usual kinetic formulations in several ways. First, we used the metabolite level (*Y*) as a surrogate for the enzymatic velocity (*v*) and the air concentration of benzene (*X*) as a surrogate for the substrate concentration, recognizing that *Y* and *X* were not strictly proportional to *v* and the substrate concentration, respectively. Second, it was necessary to include a background level (*Y*_0_) for total metabolites, to account for dietary and endogenous sources of these compounds. After fitting Equations 1 and 2 to the data, we found substantial statistical evidence (AIC difference = 5.473) favoring the model with two metabolic pathways (Equation 2). Based on the Akaike weights (Equation 3) of 0.939 and 0.061 for the two-pathway and one-pathway models, respectively, the odds are 15.4 to 1 (0.939/0.061) that Equation 2 provides a better depiction of true benzene metabolism than does Equation 1, given these data. The finding is bolstered by the independent prediction that a typical nonsmoking Chinese female exposed to non-saturating (parts per billion) levels of benzene should produce 194 μM of urinary metabolites per parts per million concentration of benzene compared with 184 μM/ppm predicted under our two-pathway model.

In preliminary analyses, we fit Equation 2 to the full set of data representing both males and females and smokers and non-smokers (*n* = 428), after adjustment for age, sex, and smoking status (data not shown). This led to unrealistically high predicted rates of benzene metabolism at low exposure levels. Upon further analysis, we attributed this result to interaction effects among sex, smoking, and the kinetic parameters, notably *X*_50,2_. Because sex and smoking were highly correlated in our Chinese subjects (most of the males were smokers and virtually all of the females were nonsmokers), we could not fully explore the interaction effect(s) and therefore focused on nonsmoking females where results were unambiguous. Future studies should seek to clarify the potential interactions of sex, smoking and benzene kinetics, using larger numbers of smoking and nonsmoking subjects of both sexes.

The affinities of the two metabolic pathways are indicated by the *K_m_* analogs, designated *X*_50,1_ and *X*_50,2_, respectively, in Table 2. [Note that factors other than *K_m_* can influence *X*_50,1_ and *X*_50,2_; see, e.g., Hildebrand et al. (1981).] The estimated values of *X*_50,1_ and *X*_50,2_ were 301 ppm and 0.594 ppm, respectively, indicating a 507-fold difference in enzyme affinities. Because the high-affinity metabolic pathway 2 would be essentially saturated at a benzene level of 1 ppm, it is perhaps not surprising that previous toxicokinetic studies, which relied primarily upon data from humans exposed to concentrations ≥1 ppm, would have failed to detect it (Bois et al. 1996; Travis et al. 1990).

Although our results favor the presence of two benzene-metabolizing pathways in humans, they provide little insight into the identities of the particular enzymes represented. Because CYP2E1 is the primary enzyme responsible for mammalian metabolism of benzene (Koop et al. 1989; Nedelcheva et al. 1999; Powley and Carlson 2000), it is reasonable to assume that the low-affinity pathway in our model (pathway 1) is dominated by CYP2E1. This conjecture is supported by previous analyses of 386 male and female subjects from our study, which showed that the homozygous variants of *CYP2E1* 1054C–T (rs2031920, ascribed to *RsaI*) had significantly lower levels of urinary muconic acid, phenol, and hydroquinone than did homozygous wild types (Kim et al. 2007). Furthermore, the ratios of the metabolite levels between homozygotes (i.e., variant/variant:wild type/wild type) became much smaller as benzene air concentrations increased above 0.1 ppm, suggesting that *CYP2E1* 1054C–T is active primarily at higher concentrations.

Regarding the identity of the high-affinity pathway 2, we speculate that this could represent another CYP enzyme(s). Of the various candidates that have been shown to oxidize benzene in mammalian *in vitro* systems, only CYP2E1, CYP2B1, and CYP2F1 appear to be active in humans, and these have apparent *K_m_* values in the order 2F1 < < 2E1 < 2B1 (Koop et al. 1989; Nedelcheva et al. 1999; Powley and Carlson 2000). Thus, although CYP2B1 could contribute to metabolic pathway 1 in our model, CYP2F1 could contribute to metabolic pathway 2, which operates at low benzene concentrations. Powley and Carlson (2000) concluded that CYP2F1 is expressed in human microsomes from both liver and lung and is particularly active in the lung, where benzene is absorbed after inhalation. Another promising candidate CYP enzyme that is worth considering in this context is CYP2A13, which is highly expressed in the human lung and has recently been shown to catalyze several known CYP2E1 substrates, namely, toluene, styrene, chlorzoxazone, and *p*-nitrophenol, with much higher affinity compared with CYP2E1 (Fukami et al. 2008). Thus, CYP2F1 and CYP2A13 should be investigated as potential contributors to the enhanced benzene metabolism observed at air concentrations < 1 ppm.

**Figure 2:**
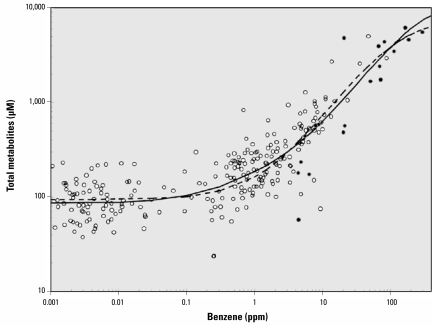
Scatterplot of levels of total metabolites versus the level of benzene in air for 263 nonsmoking female subjects. Open data points represent subjects from Tianjin, and solid points represent subjects from Shanghai. The dashed curve represents the benzene metabolite level predicted under the one-pathway model (Equation 1), and the solid curve represents the benzene metabolite level predicted under the two-pathway model (Equation 2).

**Figure 3:**
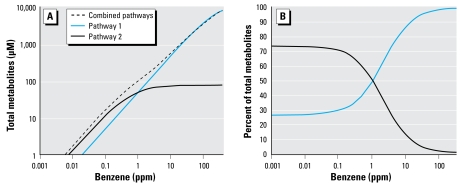
Predicted contributions of two metabolic pathways toward production of total metabolites at different levels of benzene exposure. (*A*) Predicted mean trend (metabolic pathway 1 + metabolic pathway 2) and partial trends (metabolic pathway 1 or metabolic pathway 2) representing background-adjusted levels of benzene metabolites for a typical nonsmoking female subject. Predictions were based on parameters estimated for the two-pathway model (Equation 2) from the original regression (see Table 2). (*B*) Predicted percentages of benzene metabolites from the trends shown in (*A*) for the individual metabolic pathways.

Because concentrations of benzene in ambient air tend to be < 0.01 ppm throughout the world (Kim et al. 2006a), our results suggest that the high-affinity pathway 2 is responsible for most metabolism of this airborne carcinogen in the general population (e.g., about 73% in nonsmoking females; Figure 2). Given the potential importance of benzene as a source of leukemia and other hematopoietic malignancies in the general population, it is important that our results be independently verified and that the putative high-affinity pathway 2 be identified. Nonetheless, based on the prediction of 184 μM of benzene metabolites per part per million benzene under our two-pathway model versus a prediction of 68.6 μM/ppm under our one-pathway model, it is reasonable to conclude that current risk assessments would likely underestimate leukemia risks at ambient air concentrations of benzene by a factor of about 3 for nonsmoking women.

In summary, this study of benzene exposures and metabolite levels among 263 non-smoking women provides strong statistical evidence that an unknown high-affinity pathway is responsible for most metabolism of benzene at sub-part per million air concentrations. Because benzene is a ubiquitous air contaminant that must be metabolized in order to exert toxicity, our results suggest that the metabolism of benzene and its associated leukemia risk could be substantially greater than is currently thought in the general population.

## Figures and Tables

**Table 1:** Summary statistics for the study population (nonsmoking female subjects, *n* = 263).

Statistic	Median value (range)	10th–90th percentiles
Benzene exposure (ppm)	0.644 (1.5 × 10^−4^ to 299)	0.002–8.97
Age (years)	32 (18–52)	21–44
Body mass index (kg/m^2^)	21.8 (15.4–38.2)	18.5–26.7
Weight (kg)	57 (39–96)	48–70

**Table 2:** Parameter estimates for models of benzene metabolites (corresponding to Equations 1 and 2) for 263 nonsmoking female subjects.

			Bootstrap resampling (*n* = 1,000)	
Model	Parameter	Original model estimate (SE)	Median	2.5th, 97.5th percentiles
One pathway (Equation 1)	*Y*_0_ (μM)	94.64 (4.774)	94.68	85.76, 104.1
	*Y*_max_ (μM)	8,253 (2,877)	8,252	4,602, 15,169
	*X*_50_ (ppm)	120.3 (47.49)	119.7	58.76, 251.6
	*Y*_max_:*X*_50_ (μM/ppm)	68.60	69.08	56.01, 84.84
Two pathways (Equation 2)	*Y*_0_ (μM)	87.20 (5.244)	86.30	60.13, 95.94
	*Y*_max,1_ (μM)	14,637 (10,321)	14,742	7,554, 60,992
	*X*_50,1_ (ppm)	300.6 (260.6)	301.2	121.5, 1,743
	*Y*_max,2_ (μM)	80.06 (67.26)	77.75	32.17, 209.6
	*X*_50,2_ (ppm)	0.5938 (0.9616)	0.5756	0.0030, 2.765
	*Y*_max,1_:*X*_50,1_ (μM/ppm)	48.69	48.91	32.98, 68.23
	*Y*_max,2_:*X*_50,2_ (μM/ppm)	134.8	142.7	45.84, 18,672

Abbreviations: *Y*, level of total metabolites (μM), *X*, benzene air concentration (ppm); *Y*_0_, background level of *Y; Y*_max,_*_i_*, maximum value of *Y* given the *i*th metabolic pathway; *X*_50,_*_i_*, *X* corresponding to 50% of *Y*_max,_*_i_*.
